# Homodimerization of APOBEC3G is required for inhibition of Alu retrotransposition

**DOI:** 10.1186/1742-4690-8-S2-P67

**Published:** 2011-10-03

**Authors:** Yukie Iwabu, Juan F Arias, Masaru Yokoyama, Hironori Sato, Tetsutaro Sata, Kenzo Tokunaga

**Affiliations:** 1 Department of Pathology, National Institute of Infectious Diseases, Tokyo, 162-8640, Japan; 2 Pathogen Genomics Center, National Institute of Infectious Diseases, Tokyo, 162-8640, Japan

## Background

Alu elements are active non-LTR retrotransposons that account for approximately 11 % of the human genome and are known to be not only the cause of genetic disease in germinal cells, but have also been implicated on the development of B-cell lymphomas suggesting they affect the genome of somatic cells as well. In the present study, the molecular mechanisms of the inhibitory activity of human APOBEC3 (A3) family members on Alu retrotransposition were investigated.

## Materials and methods

A3 family proteins were tested in a neomycin-resistant (neo^r^)-based retrotransposition assay. Briefly, HeLa cells were cotransfected with a neo^r^-Alu reporter vector, LINE-1 ORF2 vector and the respective A3 expression plasmids. Seventy-two hours post-transfection, G418 was added for resistance selection, and after 14 days resultant G418-resistant colonies were fixed, stained with crystal violet and counted. A series of APOBEC3G (A3G) mutants bearing deletions in the N-terminus in multiples of 30 amino acids (aa) were constructed to evaluate the responsible region. Likewise, dimerization and deamination deficient A3G mutants were also created to examine the inhibitory effect of A3G on Alu retrotransposition.

## Results

The neo^r^-based retrotransposition assay showed that all hA3 family proteins differentially inhibited Alu retrotransposition. By the deletion analyses based on A3G that is the most well-characterized A3 family member, the N-terminal 30 aa of A3G was found to determine its inhibitory activity on Alu retrotransposition. Mutational analyses showed that the inhibitory activity of A3G was independent of its deaminase activity, but dependent on its dimerization, which was found to be affected by deletion in its N-terminal 30 aa. Importantly, the dimerization of A3G was also required for the inhibition of LINE-1 retrotransposition.

## Conclusions

The N-terminal 30 aa of A3G are essential for its inhibitory activity on Alu retrotransposition, and this correlates with A3G homodimerization. The structural basis for the effect of the N-terminal 30 aa region of A3G on its dimerization is currently under investigation.

